# Surgical Margin Affects the Long-Term Prognosis of Patients With Hepatocellular Carcinoma Undergoing Radical Hepatectomy Followed by Adjuvant TACE

**DOI:** 10.1093/oncolo/oyad088

**Published:** 2023-04-08

**Authors:** Shilei Bai, Pinghua Yang, Jianwei Liu, Hui Xue, Yong Xia, Fuchen Liu, Zhao Yang, Lei Zhang, Yeye Wu, Feng Shen, Kui Wang

**Affiliations:** Department of Hepatic Surgery II, The Eastern Hepatobiliary Surgery Hospital, Naval Medical University, Shanghai, People’s Republic of China; Department of Biliary Surgery IV, The Eastern Hepatobiliary Surgery Hospital, Naval Medical University, Shanghai, People’s Republic of China; Department of Hepatic Surgery II, The Eastern Hepatobiliary Surgery Hospital, Naval Medical University, Shanghai, People’s Republic of China; Department of Hepatic Surgery II, The Eastern Hepatobiliary Surgery Hospital, Naval Medical University, Shanghai, People’s Republic of China; Department of Hepatic Surgery IV, The Eastern Hepatobiliary Surgery Hospital, Naval Medical University, Shanghai, People’s Republic of China; Department of Hepatic Surgery III, The Eastern Hepatobiliary Surgery Hospital, Naval Medical University, Shanghai, People’s Republic of China; Department of Hepatic Surgery II, The Eastern Hepatobiliary Surgery Hospital, Naval Medical University, Shanghai, People’s Republic of China; Department of Hepatic Surgery II, The Eastern Hepatobiliary Surgery Hospital, Naval Medical University, Shanghai, People’s Republic of China; Department of Hepatic Surgery II, The Eastern Hepatobiliary Surgery Hospital, Naval Medical University, Shanghai, People’s Republic of China; Department of Hepatic Surgery IV, The Eastern Hepatobiliary Surgery Hospital, Naval Medical University, Shanghai, People’s Republic of China; Department of Hepatic Surgery II, The Eastern Hepatobiliary Surgery Hospital, Naval Medical University, Shanghai, People’s Republic of China

**Keywords:** surgical margin, adjuvant TACE, hepatocellular carcinoma, prognosis, propensity score matching

## Abstract

**Background:**

The aim of this study was to investigate whether postoperative adjuvant transcatheter arterial chemoembolization (TACE) treatment in wide- and narrow-margin groups could improve the long-term prognosis of patients with hepatocellular carcinoma (HCC).

**Materials and Methods:**

A total of 670 patients with HCC who underwent radical hepatectomy from January 2016 to December 2017 were enrolled, including 397 patients and 273 patients in the wide- and narrow-margin groups. Recurrence-free survival (RFS) and overall survival (OS) outcomes were compared in the wide-margin and narrow-margin groups with and without adjuvant TACE postoperatively, respectively. Propensity score matching (PSM) analysis was used to match patients between TACE and no TACE groups in a 1:1 ratio.

**Results:**

The wide-margin resection was associated with better RFS and OS rates than narrow-margin resection for patients with HCC. Patients with postoperative adjuvant TACE had a better RFS and OS than patients without postoperative adjuvant TACE in the narrow-margin group and reduced the intrahepatic recurrence rate (39.1% vs. 52.6%, *P* = .036) and the local recurrence rate in the liver (11.2% vs. 21.4%, *P* = .032). But postoperative adjuvant TACE did not alter recurrence and survival outcomes in the wide-margin group. Similar results were noted after propensity score matching (PSM).

**Conclusion:**

The wide-margin resection had better RFS and OS than the narrow-margin resection for patients with HCC. Postoperative adjuvant TACE was associated with reduced recurrence and improved OS after narrow-margin resection, but was not effective in the wide-margin resection.

## Teaser

This study investigated whether postoperative adjuvant transcatheter arterial chemoembolization (TACE) treatment in wide- and narrow-margin groups could improve the long-term prognosis of patients with hepatocellular carcinoma.

**Implications for Practice**

For patients with HCC with tumors adjacent to large vessels or poor liver function, narrow-margin resection may be the more appropriate surgical approach, as it preserves more liver tissue and avoids damage to important vessels, but it also leads to a greater risk of recurrence and poorer long-term survival outcomes in these patients, whether postoperative anti-recurrence therapy is available for this group of patients remains unclear. The aim of this study was to analyze the prognostic differences between patients with wide and narrow margins who underwent hepatectomy with or without adjuvant TACE to guide rational clinical decision-making.

## Introduction

Hepatocellular carcinoma (HCC) is the 6th most malignant and 3rd most lethal tumor in the world.^1^ Hepatectomy is still the first option for curing early-stage HCC, but postoperative recurrence rates are as high as 70%-80%, resulting in unsatisfactory long-term survival outcomes.^2,3^ Anti-recurrence therapy after hepatectomy is currently thought to reduce the recurrence rate and prolong survival in patients, with treatment modalities including adjuvant transcatheter arterial chemoembolization (TACE), sorafenib, Huaier granules, interferon, and postoperative antiviral therapy for hepatitis B-related HCC.^4-8^

TACE is the most commonly used treatment for mid-stage HCC. TACE reduces blood flow to the tumor and induces ischemic necrosis by injecting chemotherapeutic agents and embolic agents through the arteries. Some studies suggest that adjuvant TACE after radical hepatectomy is a feasible approach that can significantly reduce recurrence and improve long-term prognosis in patients with HCC.^5,9-14^ However, there are conflicting arguments that adjuvant TACE does not improve the long-term prognosis of patients with HCC.^15^ Therefore, it has been suggested that adjuvant TACE therapy only benefits patients with HCC at high risk of recurrence, such as those with microvascular invasion (MVI), portal vein tumor thrombus (PVTT), multiple tumors, or a large HCC.^10-14,16^ It is known that, in addition to the tumor characteristics themselves, the difference in surgical approach (anatomical or non-anatomical resection, wide or narrow-margin) is also an important risk factor of recurrence in patients with HCC.^17-19^ However, current studies of adjuvant TACE are limited to tumor characteristics per se and do not consider the impact of differences in surgical approach.

Wide and narrow surgical margins are one of the most important factors affecting the long-term prognosis of patients with HCC, with patients with narrow margins having a higher chance of recurrence and worse overall survival (OS).^17,19^Previous studies have documented that most portal vein invasion and intrahepatic micrometastases are found within 10 mm of the main tumor, and rarely more than 20 mm from the tumor, thus, these authors concluded that a minimum surgical margin width of 10 mm was required.^20-22^Postoperative adjuvant TACE may be one way to improve the prognosis of patients with HCC with narrow margin, we, therefore, designed this project to investigate the safety and efficacy of postoperative adjuvant TACE in patients with wide and narrow margins through a retrospective analysis of previous cases.

## Materials and Methods

### Study Design

This study included 670 patients with HCC who underwent radical hepatectomy from January 2016 to December 2017 at the Third Affiliated Hospital of Naval Military Medical University (Eastern Hepatobiliary Surgery Hospital) on a continuous basis, including 397 patients in the wide-margin group and 273 patients in the narrow margin group. The wide-margin group included 211 patients with postoperative adjuvant TACE and 186 patients with HCC not treated with postoperative adjuvant TACE, and the narrow-margin group included 161 with and 112 patients without postoperative adjuvant TACE for HCC. The inclusion criteria were as follows: (1) postoperative pathological diagnosis of single HCC; (2) Child–Pugh classification of stage A or B7; (3) no preoperative antitumor therapy and no history of other tumors; (4) surgical approach of radical resection (R0), defined as complete removal of macroscopic tumor tissue and negative tumor margins^23^; (5) no macrovascular or biliary invasion, that is, combined bile duct, hepatic vein, or portal vein cancer thrombosis; and (6) ECOG score of less than or equal to 2. The exclusion criteria were as follows: (1) incomplete clinical data; (2) tumor recurrence within 1 month; (3) abnormal preoperative AFP not reduced to normal within 1 month postoperatively; and (4) postoperative combined serious complications not recovered within 1 month. The study was approved by the hospital ethics committee, and informed consent to use their data for scientific research was obtained from each patient.

### Preoperative Assessment and Hepatectomy

Preoperative tests included liver function, renal function, coagulation, alpha-fetoprotein (AFP), HBV and HCV antigen/antibody, HBV deoxyribonucleic acid (HBV-DNA), routine ECG, further dynamic ECG, and cardiac ultrasound if the patient had associated cardiac disease, and imaging, including chest X-ray or noncontrast computed tomography (CT), abdominal ultrasound, enhanced CT, or enhanced MRI of the upper abdomen. Preoperative diagnosis was determined according to the American Association for the Study of Liver Diseases (AASLD) criteria.^24^

All procedures were conventional open surgery with routine use of intraoperative ultrasound, and the extent of resection was based on tumor size, location, and the general condition of the patient. Major hepatectomy was defined as resection of 3 or more liver segments, and minor hepatectomy was defined as resection of less than 3 liver segments.^25^Wide or narrow margins were defined as the shortest distance from the tumor margin to the plane of resection ≥1 cm or <1 cm, depending on the postoperative pathology report, which is consistent with the approach reported elsewhere.^26,27^MVI was defined as microscopic tumor invasion as identified in the portal or hepatic veins in the surrounding liver tissues contiguous to the tumor.^28^

### Postoperative TACE

Approximately 1 month after hepatectomy, when liver function has recovered, a hepatic artery catheter is placed through the femoral artery into the innominate hepatic artery using the Seldinger technique. A hepatic angiogram is performed to detect the presence of the residual tumor in the residual liver, and TACE is performed on the whole residual liver or the corresponding half of the liver. An emulsion of doxorubicin hydrochloride (10 mg), pirarubicin or pharmorubicin (20-40 mg), and lipiodol (2-10 mL) (Lipiodol Ultrafluide, Guerbet, Aulnay-Sous-Bois, France) was then infused through the catheter. The dose of lipiodol and doxorubicin is determined by body surface area and basal liver function.

### Postoperative Follow-Up

Patients are followed up every 2 months for the first 6 months and every 6 months thereafter, and this follow-up includes testing for the tumor marker AFP in peripheral blood and ultrasound, as well as in enhanced CT or magnetic resonance imaging (MRI) of the abdomen. When tumor recurrence or metastasis is suspected, a repeat upper abdominal enhancement CT or enhancement MRI will be performed, and a recurrence or metastasis in the liver will be considered if typical signs of liver cancer are found in both imaging studies. If necessary, PET-CT or bone scans will be performed to confirm the diagnosis. Patients who had developed recurrence were treated with a multidisciplinary approach, which included rehepatectomy, TACE, radiotherapy, sorafenib, or conservative treatment. The endpoints are OS and recurrence-free survival (RFS). OS was calculated from the date of liver resection to the date of death or the date of the last follow-up visit, and RFS was calculated from the date of liver resection to the date of the first HCC recurrence or the date of the last follow-up visit and patterns of recurrence that included early recurrence (≤24 months post-treatment), type of recurrence (intra-, extra-, or synchronous intra- and extrahepatic recurrences), and site of intrahepatic recurrence. Recurrence located within 2 cm of the resection margin or ablation zone was defined as local recurrence.^29^

### Statistical Analysis

The software used for statistical analysis was R software, version 4.0.0 (https://www.r-project.org/), and categorical variables were expressed as numbers (*n*) or proportions (%). Categorical variables were compared using the *X*^2^ test or Fisher’s exact test, as appropriate. A one-to-One PSM was used to balance the differences in baseline clinicopathological features between the TACE and non-TACE groups. Covariates included in the PSM analysis were HBV-DNA (≤2000/>2000 IU/mL), prothrombin time (≤13/>13 S), platelet (≤100/>100 × 109/mL), cirrhosis(no/yes) in the wide-margin group and Child-Pugh (A/B7), platelet (≤100/>100 × 109/mL), type of operation (minor/major), microvascular invasion(negative/positive), cirrhosis(no/yes) in the narrow-margin group to calculate the propensity score by logistic regression. PSM was performed using the closest match method and the pairs on the propensity-score logit were then matched to within a range of 0.2 of standard deviation. OS and RFS were calculated by the Kaplan-Meier method generated by the log-rank test. Independent risk factors for OS and RFS were determined using univariate and multivariable Cox regression analyses. Multivariable Cox regression models with positive stepwise variable selection were used for variables with *P* < .05 in the univariate analysis. In all analyses, the level of statistical significance was set at *P* < .05.

## Results

### Patient Characteristics

**Figure 1.**
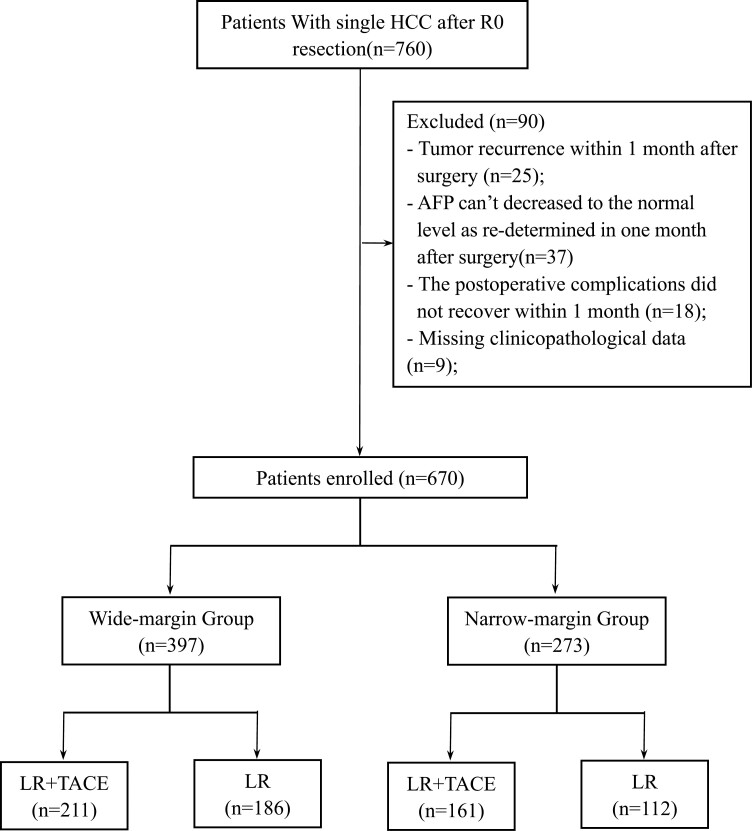
Flow chart of patient’s inclusion.

Patient inclusion and exclusion criteria are shown in Fig. 1. Of the 670 patients with HCC included, 397 were in the wide-margin group, and 273 were in the narrow-margin group. In the wide-margin group, 211 patients were treated with adjuvant TACE, and 186 patients were not treated with adjuvant TACE. Patients not treated with adjuvant TACE, compared with those treated with adjuvant TACE, had a higher proportion of HBV-DNA > 2000 IU/mL (28.0% vs. 17.1%, *P* = .013), prothrombin time (PT) > 13 s (20.4% vs. 9.95%, *P* = .005), platelets (PLT) ≤100 * 10^9^/mL (26.3% vs. 11.8%, *P* < .001) and cirrhosis (77.4% vs. 66.8%). In the narrow-margin group, the number of patients who did and did not receive postoperative adjuvant TACE treatment was 112 and 161, respectively. Patients not treated with adjuvant TACE, compared with those treated with adjuvant TACE, had a higher proportion of Child–Pugh stage B7 (5.36% vs. 0.62%, *P* = .020), PLT ≤100 * 10^9^/mL (21.4% vs. 9.94%, *P* = .014), major resection (70.5% vs. 54.0%, *P* = .009), positive MVI (49.1% vs. 32.9%, *P* = .010), and cirrhosis (77.7% vs. 65.2%, *P* = .037) (Table 1). To minimize bias due to baseline differences, we included 2 separate groups of differential variables for PSM at 1:1 by the closest match method, including 114 patients each with and without TACE in the wide-margin group and 70 patients each with and without TACE in the narrow-margin group after PSM (Table 2).

**Table 1.** Basal clinicopathological characteristics of patients with HCC with and without adjuvant TACE.

Variable	Wide margin					Narrow margin					SD^*^	*P* ^*^
	Total (*n* = 397)	No TACE (*n* =186)	TACE (*n* = 211)	*SD*	*P*	Total (*n* = 273)	No TACE (*n* = 112)	TACE (*n* = 161)	SD	*P*		
Age, year				0.005	1.000				0.119	.419	0.081	.351
≤60	324(81.6)	152 (81.7)	172 (81.5)			214(78.4)	91 (81.2)	123 (76.4)				
>60	73(18.4)	34 (18.3)	39 (18.5)			59(21.6)	21 (18.8)	38 (23.6)				
Gende**r**				0.124	.224				0.124	.404	0.006	.966
Male	344(86.9)	157 (84.4)	187 (89.0)			236(86.4)	94 (83.9)	142 (88.2)				
Female	52(13.1)	29 (15.6)	23 (11.0)			37(13.6)	18 (16.1)	19 (11.8)				
Diabetes				0.118	.335				0.064	.789	0.029	.838
No	375(94.5)	173 (93.0)	202 (95.7)			256(93.8)	104 (92.9)	152 (94.4)				
Yes	22(5.54)	13 (6.99)	9 (4.27)			17(6.23)	8 (7.14)	9 (5.59)				
Child-Pugh				0.031	1.000				0.281	.020	0.095	.244
A	392(98.7)	184 (98.9)	208 (98.6)			266(97.4)	106 (94.6)	160 (99.4)				
B7	5(1.26)	2 (1.08)	3 (1.42)			7(2.56)	6 (5.4)	1 (0.6)				
HBsAg				0.055	.694				0.070	.698	0.017	.923
Negative	53(13.4)	23 (12.4)	30 (14.2)			38(13.9)	14 (12.5)	24 (14.9)				
Positive	344(86.6)	163 (87.6)	181 (85.8)			235(86.1)	98 (87.5)	137 (85.1)				
HBV-DNA, IU/mL				0.263	.013				0.095	.532	0.082	.343
≤2000	309(77.8)	134 (72.0)	175 (82.9)			203(74.4)	86 (76.8)	117 (72.7)				
>2000	88(22.2)	52 (28.0)	36 (17.1)			70(25.6)	26 (23.2)	44 (27.3)				
TBIL, μmol/L				0.029	.862				0.040	.852	0.043	.649
≤17	294(74.1)	139 (74.7)	155 (73.5)			197(72.2)	82 (73.2)	115 (71.4)				
>17	103(25.9)	47 (25.3)	56 (26.5)			76(27.8)	30 (26.8)	46 (28.6)				
ALB, g/L				0.146	.221				0.046	.889	0.107	.226
≤35	19(4.79)	12 (6.45)	7 (3.32)			20(7.33)	9 (8.04)	11 (6.83)				
>35	378(95.2)	174 (93.5)	204 (96.7)			253(92.7)	103 (92.0)	150 (93.2)				
ALT, U/L				0.098	.389				0.085	.571	0.204	.012
≤44	270(68.0)	122 (65.6)	148 (70.1)			159(58.2)	68 (60.7)	91 (56.5)				
>44	127(32.0)	64 (34.4)	63 (29.9)			114(41.8)	44 (39.3)	70 (43.5)				
PT, S				0.295	.005				0.157	.259	0.102	.228
≤13	338(85.1)	148 (79.6)	190 (90.0)			222(81.3)	87 (77.7)	135 (83.9)				
>13	59(14.9)	38 (20.4)	21 (9.95)			51(18.7)	25 (22.3)	26 (16.1)				
NLR				0.032	.829				0.141	.309	0.219	.007
≤2.5	270(68.0)	125 (67.2)	145 (68.7)			157(57.5)	69 (61.6)	88 (54.7)				
>2.5	127(32.0)	61 (32.8)	66 (31.3)			116(42.5)	43 (38.4)	73 (45.3)				
PLT, *10^9^/mL				0.375	<.001				0.320	.014	0.107	.213
≤100	74(18.6)	49 (26.3)	25 (11.8)			40(14.7)	24 (21.4)	16 (9.94)				
>100	323(81.4)	137 (73.7)	186 (88.2)			233(85.3)	88 (78.6)	145 (90.1)				
AFP, ng/mL				0.093	.411				0.114	.427	0.116	.164
≤400	159(40.1)	79 (42.5)	80 (37.9)			94(34.4)	35 (31.2)	59 (36.6)				
>400	238(59.9)	107 (57.5)	131 (62.1)			179(65.6)	77 (68.8)	102 (63.4)				
Type of operation				0.158	.142				0.345	.009	0.376	<.001
Minor	229(57.7)	115 (61.8)	114 (54.0)			107(39.2)	33 (29.5)	74 (46.0)				
Major	168(42.3)	71 (38.2)	97 (46.0)			166(60.8)	79 (70.5)	87 (54.0)				
Transfusion				0.082	.526				0.230	.084	0.253	.002
No	360(90.7)	171 (91.9)	189 (89.6)			224(82.1)	86 (76.8)	138 (85.7)				
Yes	37(9.32)	15 (8.06)	22 (10.4)			49(17.9)	26 (23.2)	23 (14.3)				
Tumor diameter, cm				0.199	.063				0.222	.096	0.493	<.001
≤5	262(66.0)	132 (71.0)	130 (61.6)			115(42.1)	40 (35.7)	75 (46.6)				
>5	135(34.0)	54 (29.0)	81 (38.4)			158(57.9)	72 (64.3)	86 (53.4)				
Microvascular invasion				0.140	.203				0.334	.010	0.208	.010
Negative	279(70.3)	137 (73.7)	142 (67.3)			165(60.4)	57 (50.9)	108 (67.1)				
Positive	118(29.7)	49 (26.3)	69 (32.7)			108(39.6)	55 (49.1)	53 (32.9)				
Tumor capsule				0.018	.943				0.129	.362	0.234	.004
Complete	243(61.2)	113 (60.8)	130 (61.6)			197(72.2)	77 (68.8)	120 (74.5)				
Incomplete	154(38.8)	73 (39.2)	81 (38.4)			76(27.8)	35 (31.2)	41 (25.5)				
Edmondson-Steiner grade				0.128	.244				0.191	.164	0.055	.537
I-II	122(30.7)	63 (33.9)	59 (28.0)			77(28.2)	26 (23.2)	51 (31.7)				
III-VI	275(69.3)	123 (66.1)	152 (72.0)			196(71.8)	86 (76.8)	110 (68.3)				
**Cirrhosis**				0.238	.026				0.279	.037	0.055	.747
No	112(28.2)	42 (22.6)	70 (33.2)			81(29.7)	25 (22.3)	56 (34.8)				
Yes	285(71.8)	144 (77.4)	141 (66.8)			192(70.3)	87 (77.7)	105 (65.2)				

^*^SD values and *P*-values for the comparison between wide-margin and narrow-margin HCC groups.

Bold values indicate statistical significance (*P* < .05).

Abbreviations: AFP, alpha fetoprotein ALB, albumin; ALT, alanine aminotransferase; HBV-DNA, hepatitis B virus-deoxyribonucleic acid; HCC, hepatocellular carcinoma; NLR, neutrophil-to-lymphocyte ratio; PLT, platelet; PSM, propensity score matching; PT, prothrombin time; SD, standardized differences; TACE, transcatheter arterial chemoembolization; TBIL, total bilirubin.

**Table 2.** Basal clinicopathological characteristics of patients with HCC with and without adjuvant TACE after PSM.

Variable	Wide margin				Narrow margin			
	No TACE (*n* =114)	TACE (*n* = 114)	SD	*P*	No TACE (*n* = 70)	TACE (*n* = 70)	SD	*P*
Age, Year			0.023	1.000			0.103	.684
≤60	94 (82.5)	93 (81.6)			56 (80.0)	53 (75.7)		
>60	20 (17.5)	21 (18.4)			14 (20.0)	17 (24.3)		
Gender			<0.001	1.000			0.120	.636
Male	98 (86.0)	98 (86.7)			58 (82.9)	61 (87.1)		
Female	16 (14.0)	15 (13.3)			12 (17.1)	9 (12.9)		
Diabetes			0.134	.756			0.049	1.000
No	111(97.3)	113 (99.1)			63 (90.0)	64 (91.4)		
Yes	3(2.7)	1 (0.88)			7 (10.0)	6 (8.57)		
Child-Pugh			<0.001	1.000			—	—
A	113 (99.1)	113 (99.1)			70 (100)	70 (100)		
B7	1 (0.88)	1 (0.88)			0 (0)	0 (0)		
HBsAg			<0.001	1.000			0.042	1.000
Negative	16 (14.0)	16 (14.0)			10 (14.3)	9 (12.9)		
Positive	98 (86.0)	98 (86.0)			60 (85.7)	61 (87.1)		
HBV-DNA, IU/mL			0.022	1.000			0.183	.078
≤2000	91 (79.8)	92 (80.7)			55 (78.6)	45 (64.3)		
>2000	23 (20.2)	22 (19.3)			15 (21.4)	25 (35.7)		
TBIL, μmol/L			0.168	.269			0.034	1.000
≤17	92 (80.7)	84 (73.7)			54 (77.1)	53 (75.7)		
>17	22 (19.3)	30 (26.3)			16 (22.9)	17 (24.3)		
ALB, g/L			0.095	.722			0.066	1.000
≤35	5 (4.39)	3 (2.63)			3 (4.29)	4 (5.71)		
>35	109 (95.6)	111 (97.4)			67 (95.7)	66 (94.3)		
ALT, U/L			0.135	.383			0.167	.125
≤44	84 (73.7)	77 (67.5)			40 (57.2)	31 (44.3)		
>44	30 (26.3)	37 (32.5)			30 (42.8)	39 (55.7)		
PT, S			0.172	.281			0.040	1.000
≤13	105 (92.1)	99 (86.8)			60 (85.7)	59 (84.3)		
>13	9 (7.89)	15 (13.2)			10 (14.3)	11 (15.7)		
NLR			<0.001	1.000			0.157	.176
≤2.5	77 (67.5)	77 (67.5)			41 (58.6)	32 (45.7)		
>2.5	37 (32.5)	37 (32.5)			29 (41.4)	38 (54.3)		
PLT, *10^9^/mL			0.055	.835			0.090	.791
≤100	14 (12.3)	12 (10.5)			9 (12.9)	7 (10.0)		
>100	100 (87.7)	102 (89.5)			61 (87.1)	63 (90.0)		
AFP, ng/mL			0.175	.108			<0.001	1.000
≤400	55 (48.2)	42 (36.8)			25 (35.7)	25 (35.7)		
>400	59 (51.8)	72 (63.2)			45 (64.3)	45 (64.3)		
Type of operation			0.071	.688			0.059	.861
Minor	67 (58.8)	63 (55.3)			27 (38.6)	25 (35.7)		
Major	47 (41.2)	51 (44.7)			43 (61.4)	45 (64.3)		
Transfusion			0.100	.614			0.106	.677
No	104 (91.2)	107 (93.9)			54 (77.1)	57 (81.4)		
Yes	10 (8.77)	7 (6.14)			16 (22.9)	13 (18.6)		
Tumor diameter, cm			0.019	1.000			0.059	.862
≤5	78 (68.4)	79 (69.3)			28 (40.0)	26 (37.1)		
>5	36 (31.6)	35 (30.7)			42 (60.0)	44 (62.9)		
Microvascular invasion			0.153	.315			0.116	.607
Negative	83 (72.8)	75 (65.8)			39 (55.7)	43 (61.4)		
Positive	31 (27.2)	39 (34.2)			31 (44.3)	27 (38.6)		
Tumor capsule			0.125	.420			0.064	.849
Complete	70 (61.4)	63 (55.3)			50 (71.4)	52 (74.3)		
Incomplete	44 (38.6)	51 (44.7)			20 (28.6)	18 (25.7)		
Edmondson-Steiner grade			0.112	.482			0.034	1.000
I-II	41 (36.0)	35 (30.7)			16 (22.9)	17 (24.3)		
III-VI	73 (64.0)	79 (69.3)			54 (77.1)	53 (75.7)		
Cirrhosis			0.020	1.000			0.032	1.000
No	30 (26.3)	31 (27.2)			19 (27.1)	18 (25.7)		
Yes	84 (73.7)	83 (72.8)			51 (72.9)	52 (74.3)		

Bold values indicate statistical significance (*P* < .05).

Abbreviations: AFP, alpha fetoprotein; ALB, albumin; ALT, alanine aminotransferase; HBV-DNA, hepatitis B virus-deoxyribonucleic acid; HCC, hepatocellular carcinoma; NLR, neutrophil-to-lymphocyte ratio; PLT, platelet; PSM, propensity score matching; PT, prothrombin time; SD, standardized differences; TACE, transcatheter arterial chemoembolization; TBIL, total bilirubin

### Adverse Events of TACE

Of the 372 patients treated with adjuvant TACE, 261 (70.1%) patients had associated complications, including fever, abdominal pain, nausea and vomiting, elevated transaminases, elevated bilirubin, and liver abscess. Based on the Clavien–Dindo complication grading system, 225 patients with Clavien-Dindo grade 1, 34 patients with Clavien-Dindo grade 2, and 2 patients with Clavien-Dindo grade 3, with liver abscess and biliary-related complications, which improved with liver abscess puncture and drainage and endoscopic treatment, respectively, with no complications above Clavien–Dindo grade 3 (Supplementary Table S1).

### Survival Analysis

The median follow-up time was 52.2 months in the wide-margin group and 50.0 months in the narrow-margin group. The 1-, 3-, and 5-year RFS and OS rates were better in the wide-margin group than in the narrow-margin group (72.6%, 53.0%, 47.1% vs. 65.4%, 37.6%, 31.8%, *P* < .001; 94.2%, 79.3%, 67.6% vs. 83.4%, 58.7%, 49.7%, *P* < .001) (Supplementary Fig. S1A, S1B). PSM was performed to minimize bias due to the difference in baseline between the 2 groups for wide and narrow margins, with 213 cases each in the wide and narrow-margin groups included after PSM (Supplementary Table S2). The 1-, 3-, and 5-year RFS and OS rates for the wide-margin and narrow-margin groups were 72.1%, 53.4%, 47.1% vs. 67.9%, 40.7%, 34.9% and 95.1%, 77.1%, 64.3% vs. 86.3%, 64.6%, 54.8%, respectively, with *P* values of 0.026 and 0.005. Recurrence and OS outcomes were better in the wide-margin group than in the narrow-margin group (Supplementary Fig. S1C, S1D).

**Figure 2.**
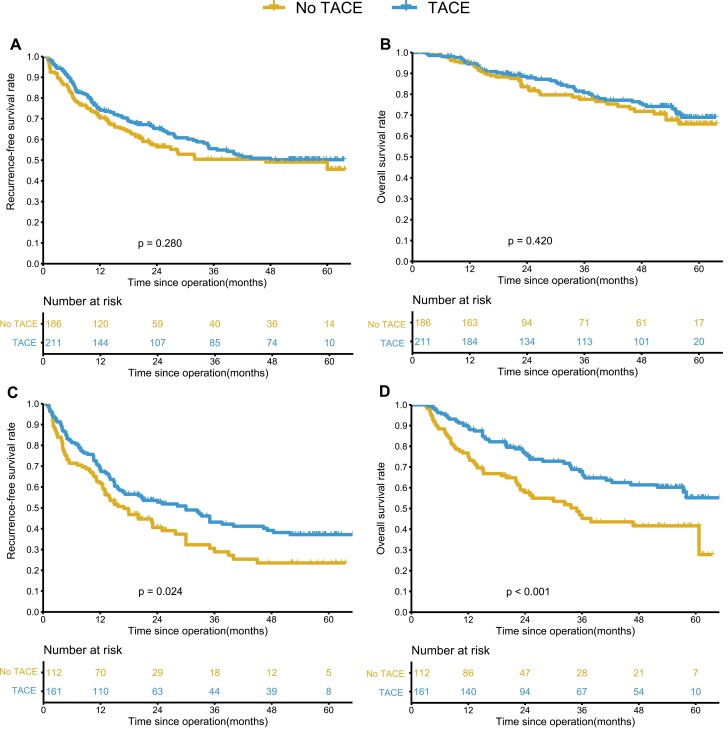
Kaplan-Meier analysis of recurrence-free survival (RFS) and overall survival (OS) for hepatocellular carcinoma (HCC) patients after liver resection with or without adjuvant TACE before PSM. (**A-B**) In the wide-margin group, tumor recurrence and OS for patients with and without adjuvant TACE. (**C-D**) In the narrow-margin group, tumor recurrence and OS for patients with and without adjuvant TACE.

The TACE and no TACE groups had similar 1-, 3-, and 5-year RFS and OS rates in the wide-margin group (74.6%, 55.6%, 50.2% vs. 70.4%, 50.4%, 45.6%, *P* = .280; 94.6%, 80.8%, 68.9% vs. 94.5%, 77.6%, 65.8%, *P* = .420) (Fig. 2A, 2B). In the narrow-margin group, the RFS and OS rates at 1, 3, and 5 years were better in the TACE group than in the group without TACE (68.1%, 43.1%, 37.1% vs. 61.6%, 28.9%, 23.6%, *P* = .024; 89.3%, 67.8%, 55.2% vs. 75.0%, 45.2%, 41.6%, *P* < .001) (Fig. 2C, 2D).

**Figure 3.**
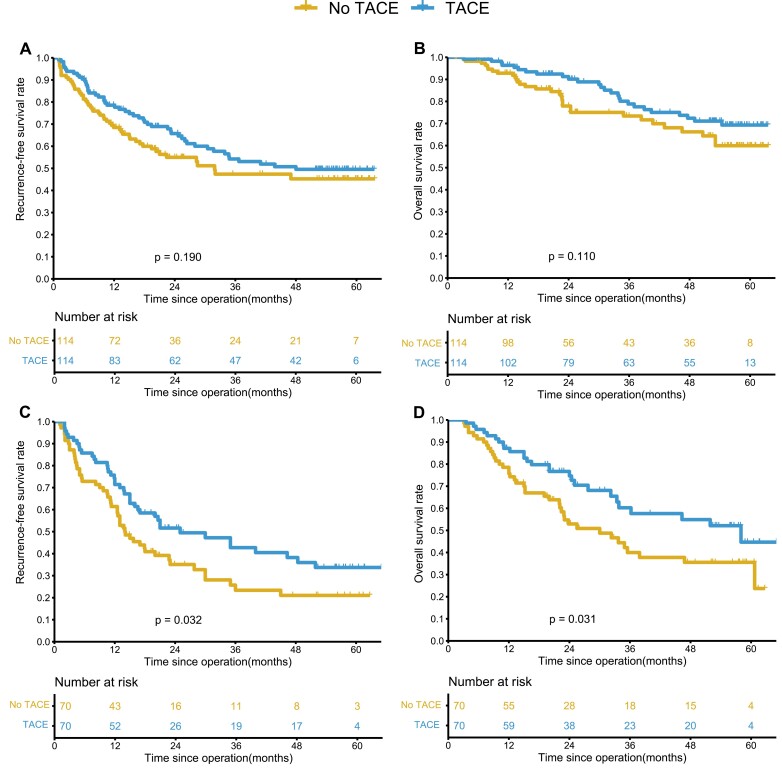
Kaplan-Meier analysis of recurrence-free survival (RFS) and overall survival (OS) for hepatocellular carcinoma (HCC) patients after liver resection with or without adjuvant TACE after PSM. (**A-B**) In the wide-margin group, tumor recurrence and OS for patients with and without adjuvant TACE. (**C-D**) In the narrow-margin group, tumor recurrence and OS for patients with and without adjuvant TACE.

A similar prognostic outcome as before PSM can be obtained after PSM. After PSM, in the wide-margin group, the TACE and no TACE groups had similar 1-, 3-, and 5-year RFS and OS rates (78.6%, 54.2%, 49.5% vs. 68.5%, 47.4%, 45.2%, *P* = .190; 95.4%, 74.7%, 69.0% vs. 92.8%, 72.7%, 59.4%, *P* = .110) (Fig. 3A, 3B). In the narrow-margin group, the RFS and OS rates at 1, 3, and 5 years were better in the TACE group than in the group without TACE (71.4%, 42.7%, 33.7% vs. 61.4%, 23.4%, 21.0%, *P* = .032; 87.1%, 60.2%, 44.7% vs. 75.7%, 40.0%, 35.5%, *P* = .031) (Fig. 3C, 3D).

### Independent Risk Factors for Tumor Recurrence and OS

The results of the univariate analysis are shown in Supplementary Tables S3 and S4. In the multivariable analysis, patients with adjuvant TACE had a lower recurrence rate and better OS than patients without TACE in the narrow-margin group (pre-PSM RFS: hazard ratio [HR]: 0.77, 95% CI, 0.56-0.95, *P* = .035; OS: 0.60, 0.40-0.89, *P* = .011; after PSM RFS: 0.65, 0.43-1.00, *P* = .048; OS: 0.59, 0.36-0.97, *P* = .037). In the wide-margin group, adjuvant TACE treatment did not improve RFS or OS, either before or after PSM (Table 3).

**Table 3.** Multivariable cox regression analysis of recurrence-free survival (RFS) and overall survival (OS) in patients with HCC with wide and narrow of margin before and after PSM.

Variable	Wide margin				Narrow margin			
	RFS		OS		RFS		OS	
	HR (95% CI)	*P*	HR (95% CI)	*P*	HR (95% CI)	*P*	HR (95% CI)	*P*
Before PSM								
Child-Pugh, B vs. A	3.47(1.25-9.66)	.017	—	—	—	—	—	—
ALT, U/L, ≥ vs. < 44	1.63(1.20-2.23)	.002	1.64(1.06-2.50)	.027	—	—	—	—
NLR, > vs. ≤2.5	—	—	—	—	1.51(1.09-2.09)	0.014	1.76(1.19-2.61)	.005
Tumor diameter, > vs. ≤5 cm	1.89(1.38-2.58)	<.001	1.97(1.28-3.05)	.002	—	—	—	—
MVI, positive vs. negative	1.88(1.36-2.60)	<.001	2.42(1.55-3.78)	<.001	1.83(1.32-2.55)	<0.001	1.87(1.25-2.80)	.002
TACE, yes vs. no	—	—	—	—	0.77(0.56-0.95)	0.035	0.60(0.40-0.89)	.011
After PSM								
HBsAg, positive vs. negative	2.15(1.07-4.31)	.031	—	—	—	—	—	—
PT, ≥ vs. <13 s	2.50(1.45-4.31)	.001	2.18(1.11-4.27)	.023	—	—	—	—
Type of operation, major vs. minor	—	—	—	—	1.77(1.06-2.96)	0.028	—	—
Transfusion, yes vs. no	—	—	—	—	—	—	1.89(1.10-3.24)	.020
Tumor diameter, > vs. ≤ 5 cm	2.27(1.50-3.43)	<.001	2.17(1.26-3.76)	.006	—	—	—	—
MVI, positive vs. negative	1.80(1.18-2.73)	.006	2.59(1.48-4.53)	.001	1.72(1.13-2.61)	0.012	1.66(1.01-2.71)	.043
TACE, yes vs. no	—	—	—	—	0.65(0.43-1.00)	0.048	0.59(0.36-0.97)	.037

Bold values indicate statistical significance (*P* < 0.05).

Abbreviations: ALT, alanine aminotransferase; HCC, hepatocellular carcinoma; NLR, neutrophil-to-lymphocyte ratio; OS, overall survival; PSM, propensity score matching; TACE, transcatheter arterial chemoembolization.

### Patterns of Recurrence After Liver Resection With Wide- And Narrow-Margin

The patterns of recurrence in patients with HCC are shown in Table 4. In the narrow-margin group, the number of recurrences was 90 (90/161, 55.9%) and 74 (74/112, 66.1%) in the TACE group and no TACE group, respectively, although there was no statistical difference between the 2 groups (*P* = .118), the proportion of recurrences was 10.2% lower in the TACE group compared with the no TACE group, and the TACE group reduced the intrahepatic recurrence rate (39.1% vs. 52.6%, *P* = .036) and the local recurrence rate in the liver (11.2% vs. 21.4%, *P* = .032) compared to the no TACE group. In the wide-margin group, the number of recurrences in the TACE and no TACE group was 92(92/211,43.6%) and 81(81/186,43.5%), respectively, which were not statistically different (*P* = 1.000). Postoperative adjuvant TACE did not improve intrahepatic and local recurrence compared to patients without adjuvant TACE (31.2% vs. 29.3%, *P* = .779; 14.0% vs. 12.8%, *P* = .843).

**Table 4.** Patterns of recurrence after liver resection with wide- and narrow-margin.

Parameters	The entire cohort (*n*, %)					
	Wide margin			Narrow margin		
	No TACE (*n* =186)	TACE (*n* = 211)	*P*	No TACE (n = 112)	TACE (*n* = 161)	*P*
No. of recurrent cases	81(43.5)	92(43.6)	1.000	74(66.1)	90(55.9)	**.118**
Time to recurrence, months						
≤24	74(39.7)	69(32.7)	.173	64(57.1)	74(46.0)	.090
Type of recurrence						
Intrahepatic	58(31.2)	62(29.3)	.779	59(52.6)	63(39.1)	**.036**
Extrahepatic	15(8.0)	18(8.6)	1.000	9(8.0)	17(10.6)	.648
Intra-plus extrahepatic	8(4.3)	11(5.2)	.849	6(5.3)	10(6.2)	.973
Site of intrahepatic recurrence^†^						
Local	26(14.0)	27(12.8)	.843	24(21.4)	18(11.2)	**.032**
Adjacent segment	19(10.2)	21(10.0)	1.000	17(15.2)	20(12.4)	.635
Distal segment	12(6.4)	15(7.1)	.953	13(11.6)	17(10.5)	.939
Multisegment	9 (4.8)	10(4.7)	1.000	11(9.8)	18(11.1)	.873
Recurrence			.280			**.024**
1-year recurrence rate, %	29.6	25.4		38.4	31.9	
3-year recurrence rate, %	49.6	44.4		71.1	56.9	
5-year recurrence rate, %	54.4	49.8		76.4	62.9	

^†^Calculated in patients with intrahepatic only and intra-plus extrahepatic recurrences. Bold values indicate statistical significance (*P* < .05).

Local recurrence, any recurrence located within 2 cm of the surgical margin, irrespective of any additional recurrence in other parts of the liver; adjacent segment recurrence, any recurrence located in the adjacent segment or in the same segment after subsegmentectomy, but with 2 cm away from the surgical margin; distal segment recurrence, any recurrence located not in the adjacent segment or in the contralateral hemiliver; multisegment recurrences, recurrences which involve multiple hepatic segments.

## Discussion

The aim of this study was to analyze the prognostic differences between patients with wide and narrow margins who underwent hepatectomy with or without adjuvant TACE to guide rational clinical decision-making. In the wide-margin group, postoperative adjuvant TACE did not improve the prognosis of patients with HCC , while in the narrow-margin group, postoperative adjuvant TACE had a lower recurrence rate and better survival outcome than the group without TACE. Similar results were obtained after PSM minimize bias, and the adverse effects of TACE treatment were generally manageable. This is the first study to examine the long-term prognostic impact of postoperative anti-recurrence therapy in patients with HCC who underwent surgery with wide and narrow margins.

Wide and narrow margins are important factors affecting the prognosis of patients with HCC .^17,26,30^ For patients with HCC with tumors adjacent to large vessels or poor liver function, narrow-margin resection may be the more appropriate surgical approach, as it preserves more liver tissue and avoids damage to important vessels, but it also leads to a greater risk of recurrence and poorer long-term survival outcomes in these patients.^30^ Liu et al. analyzed 106 patients with narrow margins and 134 patients with wide margins. Their 1-, 2-, and 3-year recurrence-free survival (RFS) and OS rates were 55.8%, 43.9%, 36.9% vs. 78.7%, 67.9%, 60.2%, and 83.5%, 65.6%, 60.6% vs. 94.0%, 89.5%, 84.2%, respectively, with *P* values of less than 0.001.^19^ In this study, we also analyzed the prognosis of 273 patients in the narrow-margin group and 397 patients in the wide-margin group with 5-year recurrence and OS rates of 68.2% vs. 52.9% and 49.3% vs. 67.4%, respectively. Both were significantly different. The study also performed PSM to minimize bias in both the wide- and narrow-margin groups, and results were obtained after PSM that were similar to those obtained before PSM, confirming studies related to the poorer prognosis of patients in the narrow-margin group. For patients at high risk of recurrence, such as those with narrow margins, postoperative prophylactic TACE may be one way to improve prognosis.

TACE reduces blood flow to the tumor and induces ischemic necrosis through arterial injection of chemotherapeutic agents and embolic agents. Postoperative TACE has been shown in several studies to improve the prognosis of patients with a high risk of HCC recurrence.^9,10,13,16,18^An RCT by Wei et al. included a randomized group of 250 patients with solitary MVI-positive HCC, 125 with postoperative TACE, and 125 with surgery alone, with 5-year tumor-free and OS rates of 26.7% vs. 22.6% and 40.2% vs. 28.8%, respectively, with *P* values of 0.020 and 0.029, which both indicate significant difference.^13^ Liu et al. analyzed 246 patients with HCC with combined portal vein thrombosis (PVTT), including 90 patients with postoperative TACE and 156 patients without postoperative TACE, and showed that tumor-free survival and OS were both better in the postoperative TACE group than in the no-TACE group.^10^ However, this type of study only considered the characteristics of the tumor itself and did not include the prognostic impact of the surgical approach.

In this study, we analyzed the prognostic impact of postoperative adjuvant TACE in the wide-margin and narrow-margin groups and showed that in the wide-margin group, which included 211 patients with HCC who underwent adjuvant TACE and 186 patients without adjuvant TACE, there was no significant prognostic impact with or without adjuvant TACE (5-year recurrence rate 49.8% vs. 54.4%, *P* = .280; 5-year survival rate of 68.9% vs. 65.8%, *P* = .420). In the narrow-margin group, which included 161 patients with and 112 patients without postoperative adjuvant TACE, postoperative prophylactic TACE treatment significantly reduced recurrence rates and prolonged patient survival (5-year recurrence rate 62.9% vs. 76.4%, *P* = .024; 5-year survival rate of 54.7% vs. 41.6%, *P* < .001). PSM analysis was performed separately for both groups to minimizing bias due to baseline differences. Similar results were obtained after PSM as before PSM, and the univariate and multivariable analyses confirmed the above. In the comparison of recurrence patterns, postoperative adjuvant TACE in the narrow-margin group reduced the intrahepatic recurrence rate (39.1% vs. 52.6%, *P* = .036) and the local recurrence rate in the liver (11.2% vs. 21.4%, *P* = .032) compared to patients without adjuvant TACE. we demonstrate that postoperative TACE is an effective treatment for patients with HCC who cannot undergo wide-margin resection to prolong survival and reduce recurrence rates, while neoadjuvant TACE may be a better treatment modality as the tumor remains in situ and can be treated more effectively. Our findings provide the rationale for neoadjuvant TACE for high-risk lesions defined by anatomical proximity to vascular structures that may preclude a wide-margin resection. We, therefore, recommend postoperative adjuvant TACE treatment only for patients with HCC with narrow margins of surgery.

The study currently is currently limited in that it is a single-center retrospective study, and a large sample of prospective studies is needed to further confirm the results. Second, the drug, dose, and frequency of adjuvant TACE may affect the prognosis of patients with HCC.

## Conclusion

Narrow margins had a higher recurrence rate and worse OS outcome than wide margins, and in the wide-margin group, the use of postoperative adjuvant TACE did not improve the prognosis of patients with HCC . In the narrow-margin group, postoperative adjuvant TACE treatment resulted in a lower recurrence rate and a better OS outcome.

## Supplementary Material

oyad088_suppl_Supplementary_Figure_S1

oyad088_suppl_Supplementary_Table_S1

oyad088_suppl_Supplementary_Table_S2

oyad088_suppl_Supplementary_Table_S3

oyad088_suppl_Supplementary_Table_S4

## Data Availability

The data underlying this article will be shared on reasonable request to the corresponding author.
