# Supplementary figures and images for: Surgical Margin Affects the Long-Term Prognosis of Patients With Hepatocellular Carcinoma Undergoing Radical Hepatectomy Followed by Adjuvant TACE
Source: Oncologist. 2023 Apr 8;28(8):e633–44. doi: 10.1093/oncolo/oyad088 (PMC10400125; doi:10.1093/oncolo/oyad088)

—+— Narrow —+— Width

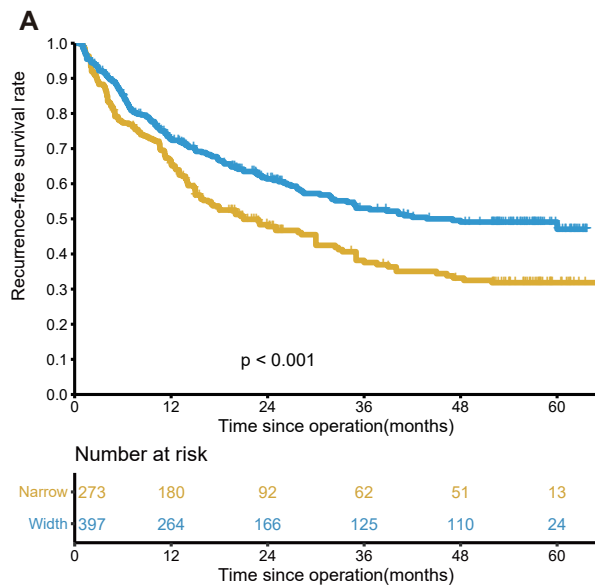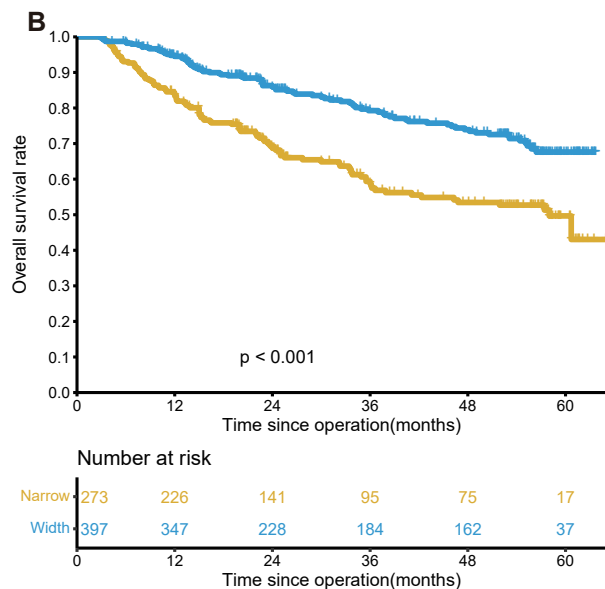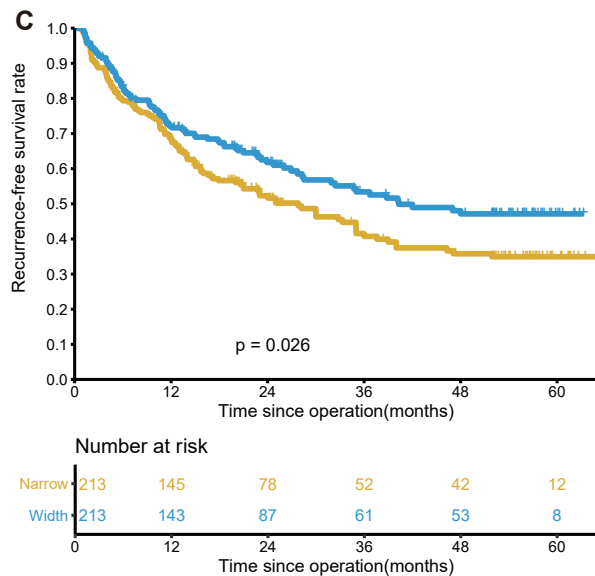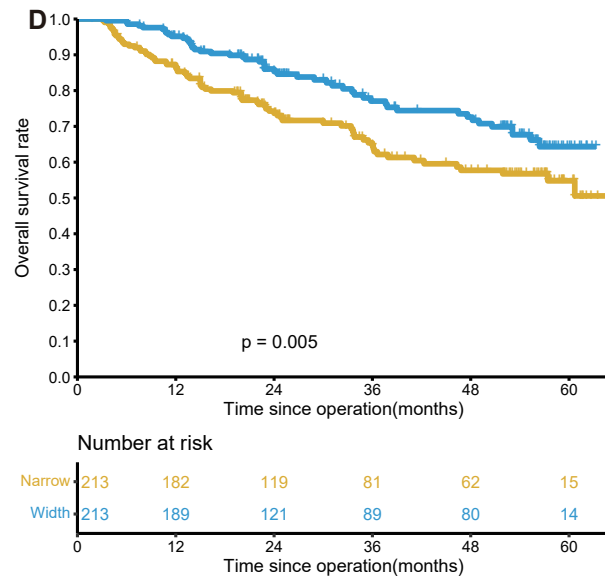

Supplement: oyad088_suppl_Supplementary_Figure_S1 [file oyad088_suppl_supplementary_figure_s1.pdf]
