# Supplementary material for: Surgical Margin Affects the Long-Term Prognosis of Patients With Hepatocellular Carcinoma Undergoing Radical Hepatectomy Followed by Adjuvant TACE
Source: Oncologist. 2023 Apr 8;28(8):e633–44. doi: 10.1093/oncolo/oyad088 (PMC10400125; doi:10.1093/oncolo/oyad088)
Supplement: oyad088_suppl_Supplementary_Table_S1 [file oyad088_suppl_supplementary_table_s1.docx]

**Supplemental online Table 1. Short-term adverse events of adjuvant transarterial chemoembolization measured by Clavien-Dindo classification**

| **Clavien-Dindo grade** | **n (%)** |
| --- | --- |
| **I** | **225(60.5)** |
| **II** | **34(9.1)** |
| **III** | **2(0.5)** |
| **IV** | **0** |
| **V** | **0** |
| **Total** | **261(70.1)** |
