# Supplementary material for: Surgical Margin Affects the Long-Term Prognosis of Patients With Hepatocellular Carcinoma Undergoing Radical Hepatectomy Followed by Adjuvant TACE
Source: Oncologist. 2023 Apr 8;28(8):e633–44. doi: 10.1093/oncolo/oyad088 (PMC10400125; doi:10.1093/oncolo/oyad088)
Supplement: oyad088_suppl_Supplementary_Table_S2 [file oyad088_suppl_supplementary_table_s2.docx]

**Supplemental online Table 2.** **Basal clinicopathological characteristics of HCC patients** **with wide and narrow margin after PSM**

| **Variable** | **n (%)** | | | |
| --- | --- | --- | --- | --- |
|  | **Narrow (n=213)** | **Width (n =213)** | ***SD*** | ***p*** |
| **Age,** Year |  |  | 0.139 | 0.188 |
| ≤60 | 163(76.5) | 175(82.2) |  |  |
| >60 | 50(23.5) | 38(17.8) |  |  |
| **Gender** |  |  | 0.069 | 0.569 |
| Male | 182(85.4) | 187(87.8) |  |  |
| Female | 31(14.6) | 26(12.2) |  |  |
| **Diabetes** |  |  | 0.038 | 0.691 |
| No | 198(93.0) | 201(94.4) |  |  |
| Yes | 15(7.04) | 12(5.63) |  |  |
| **Child-Pugh** |  |  | 0.069 | 0.724 |
| A | 208(97.7) | 210(98.6) |  |  |
| B | 5(2.35) | 3(1.41) |  |  |
| **HBsAg** |  |  | 0.081 | 0.486 |
| Negative | 33(15.5) | 27(12.7) |  |  |
| Positive | 180(84.5) | 186(87.3) |  |  |
| **HBV-DNA**, IU/mL |  |  | 0.080 | 0.478 |
| ≤2000 | 164(77.0) | 171(80.3) |  |  |
| >2000 | 49(23.0) | 42(19.7) |  |  |
| **TBIL**, μmol/L |  |  | 0.14 | 0.183 |
| ≤17 | 152(71.4) | 165(77.5) |  |  |
| >17 | 61(28.6) | 48(22.5) |  |  |
| **ALB**, g/L |  |  | 0.146 | 0.198 |
| ≤35 | 15(7.04) | 8(3.76) |  |  |
| >35 | 198(93.0) | 205(96.2) |  |  |
| **ALT**, U/L |  |  | 0.181 | 0.078 |
| ≤44 | 135(63.4) | 153(71.8) |  |  |
| >44 | 78(36.6) | 60(28.2) |  |  |
| **PT**, S |  |  | 0.141 | 0.188 |
| ≤13 | 173(81.2) | 184(86.4) |  |  |
| >13 | 40(18.8) | 29(13.6) |  |  |
| **NLR** |  |  | 0.039 | 0.762 |
| ≤2.5 | 134(62.9) | 138(64.8) |  |  |
| >2.5 | 79(37.1) | 75(35.2) |  |  |
| **PLT**, *10^9^/ml |  |  | 0.065 | 0.594 |
| ≤100 | 31(14.6) | 36(16.9) |  |  |
| >100 | 182(85.4) | 177(83.1) |  |  |
| **AFP**, ng/mL |  |  | 0.039 | 0.765 |
| ≤400 | 84(39.4) | 80(37.6) |  |  |
| >400 | 129(60.6) | 133(62.4) |  |  |
| **Type of operation** |  |  | 0.075 | 0.497 |
| Minor | 96(45.1) | 104(48.8) |  |  |
| Major | 117(54.9) | 109(51.2) |  |  |
| **Transfusion** |  |  | 0.113 | 0.308 |
| No | 182(85.4) | 190(89.2) |  |  |
| Yes | 31(14.6) | 23(10.8) |  |  |
| **Tumor diameter**, cm |  |  | 0.028 | 0.846 |
| ≤5 | 104(48.8) | 107(50.2) |  |  |
| >5 | 109(51.2) | 106(49.8) |  |  |
| **Microvascular invasion** |  |  | 0.049 | 0.688 |
| Negative | 137(64.3) | 132(62.0) |  |  |
| Positive | 76(35.7) | 81(38.0) |  |  |
| **Tumor capsule** |  |  | 0.074 | 0.513 |
| Complete | 152(71.4) | 159(74.6) |  |  |
| Incomplete | 61(28.6) | 54(25.4) |  |  |
| **Edmondson-Steiner grade** |  |  | 0.114 | 0.287 |
| I-II | 68(31.9) | 57(26.8) |  |  |
| III-VI | 145(68.1) | 156(73.2) |  |  |
| **Cirrhosis** |  |  | 0.051 | 0.676 |
| No | 64(30.0) | 69(32.4) |  |  |
| Yes | 149(70.0) | 144(67.6) |  |  |

Bold values indicate statistical significance (P < 0.05).

**Abbreviations:** HCC, Hepatocellular Carcinoma; TACE, transcatheter arterial chemoembolization; PSM: propensity score matching; SD: standardized differences; HBV-DNA, hepatitis B virus-deoxyribonucleic acid; TBIL, total bilirubin; ALB, albumin; ALT, Alanine aminotransferase; PT, Prothrombin time; NLR, neutrophil‐to‐lymphocyte ratio; PLT, platelet; AFP, alpha fetoprotein.
