# Supplementary material for: Surgical Margin Affects the Long-Term Prognosis of Patients With Hepatocellular Carcinoma Undergoing Radical Hepatectomy Followed by Adjuvant TACE
Source: Oncologist. 2023 Apr 8;28(8):e633–44. doi: 10.1093/oncolo/oyad088 (PMC10400125; doi:10.1093/oncolo/oyad088)
Supplement: oyad088_suppl_Supplementary_Table_S3 [file oyad088_suppl_supplementary_table_s3.docx]

**Supplemental online Table 3. Univariate cox regression analysis of recurrence-free survival (RFS) and overall survival (OS) in HCC patients with wide and narrow of margin before PSM**

| **Variable** | **Wide Margin** | | | |  | **Narrow Margin** | | | |
| --- | --- | --- | --- | --- | --- | --- | --- | --- | --- |
|  | **RFS** | | **OS** | |  | **RFS** | | **OS** | |
|  | **HR (95% CI)** | ***P*** | **HR (95% CI)** | ***P*** |  | **HR (95% CI)** | ***P*** | **HR (95% CI)** | ***P*** |
| **Age**, Years  > vs. ≤60 | 0.71(0.47-1.07) | 0.106 | 0.61(0.32-1.15) | 0.126 |  | 0.98(0.68-1.42) | 0.909 | 0.93(0.59-1.46) | 0.760 |
| **Gender**,  Female vs. Male | 0.71(0.44-1.15) | 0.163 | 0.73(0.38-1.41) | 0.352 |  | 0.84(0.52-1.35) | 0.464 | 1.04(0.61-1.77) | 0.878 |
| **Diabetes**  Yes vs. No | 0.65(0.31-1.39) | 0.265 | 0.83(0.30-2.26) | 0.714 |  | 1.40(0.79-2.46) | 0.246 | 1.33(0.67-2.64) | 0.411 |
| **Child-Pugh**  B7 vs. A | 3.26(1.21-8.83) | 0.020 | 1.21(0.17-8.68) | 0.851 |  | 0.43(0.11-1.72) | 0.232 | 0.76(0.19-3.09) | 0.704 |
| **HBsAg**  Positive vs. Negative | 1.75(1.06-2.89) | 0.029 | 2.56(1.12-5.88) | 0.026 |  | 1.24(0.78-1.99) | 0.360 | 1.17(0.67-2.04) | 0.592 |
| **HBV-DNA level**, IU/mL  ≥ vs. <2000 | 1.17(0.82-1.66) | 0.385 | 1.12(0.67-1.87) | 0.658 |  | 1.17(0.83-1.65) | 0.363 | 1.08(0.70-1.65) | 0.732 |
| **TBIL**,μmol/L,  ≥ vs. <17 | 0.97(0.69-1.37) | 0.857 | 0.91(0.54-1.54) | 0.731 |  | 0.67(0.46-0.96) | 0.030 | 0.67(0.43-1.05) | 0.081 |
| **ALB**, g/L,  ≥vs. < 35 | 1.14(0.53-2.42) | 0.743 | 2.25(0.55-9.13) | 0.258 |  | 1.15(0.62-2.12) | 0.659 | 1.21(0.56-2.61) | 0.621 |
| **ALT**, U/L,  ≥ vs. < 44 | 1.64(1.21-2.22) | 0.001 | 1.57(1.02-2.40) | 0.039 |  | 0.87(0.63-1.18) | 0.365 | 0.83(0.57-1.22) | 0.337 |
| **PT**, S  ≥ vs. <13 | 1.25(0.83-1.89) | 0.288 | 1.24(0.70-2.21) | 0.455 |  | 0.83(0.55-1.26) | 0.381 | 0.88(0.54-1.44) | 0.605 |
| **NLR**  > vs. ≤2.5 | 1.08(0.78-1.49) | 0.643 | 1.54(1.00-2.38) | 0.050 |  | 1.50(1.10-2.04) | 0.01 | 1.90(1.31-2.76) | 0.001 |
| **PLT**, *10^9^/ml,  > vs. ≤100 | 0.99(0.68-1.46) | 0.965 | 1.19(0.67-2.10) | 0.559 |  | 0.89(0.57-1.37) | 0.582 | 0.76(0.46-1.25) | 0.278 |
| **AFP**, μg/L,  > vs. ≤400 | 1.28(0.94-1.74) | 0.120 | 1.21(0.78-1.87) | 0.389 |  | 1.66(1.18-2.34) | 0.004 | 1.98(1.27-3.10) | 0.003 |
| **Type of operation**,  Major vs. Minor | 1.16(0.86-1.56) | 0.345 | 1.22(0.80-1.87) | 0.345 |  | 1.58(1.14-2.19) | 0.006 | 2.22(1.47-3.36) | <0.001 |
| **Transfusion**  Yes vs. No | 1.40(0.89-2.21) | 0.146 | 1.35(0.70-2.60) | 0.377 |  | 1.53(1.05-2.23) | 0.026 | 1.68(1.09-2.60) | 0.019 |
| **Tumor diameter,** cm,  > vs. ≤5 | 2.09(1.55-2.83) | <0.001 | 2.52(1.65-3.84) | <0.001 |  | 1.78(1.29-2.45) | <0.001 | 2.37(1.57-3.58) | <0.001 |
| **Microvascular invasion**  Positive vs. Negative | 2.07(1.53-2.82) | <0.001 | 3.13(2.05-4.77) | <0.001 |  | 2.10(1.54-2.85) | <0.001 | 2.59(1.78-3.76) | <0.001 |
| **Tumor capsule**,  Incomplete vs. Complete | 1.09(0.80-1.47) | 0.583 | 1.55(1.02-2.37) | 0.041 |  | 1.17(0.83-1.63) | 0.371 | 1.21(0.80-1.82) | 0.361 |
| **Edmondson-Steiner grade**,  III-VI vs. I-II | 1.46(1.04-2.06) | 0.030 | 2.16(1.26-3.71) | 0.005 |  | 1.54(1.08-2.20) | 0.017 | 2.29(1.42-3.68) | 0.001 |
| **Cirrhosis**,  Yes vs. No | 1.07(0.76-1.50) | 0.694 | 0.79(0.50-1.25) | 0.319 |  | 1.25(0.88-1.76) | 0.208 | 1.31(0.86-2.01) | 0.207 |
| **TACE**,  Yes vs. No | 0.85(0.63-1.14) | 0.275 | 0.84(0.55-1.29) | 0.427 |  | 0.7(0.51-0.95) | 0.024 | 0.53(0.37-0.77) | 0.001 |

Bold values indicate statistical significance (P < 0.05). **Abbreviation**s: OS, overall survival; PSM, propensity score matching; HCC, Hepatocellular Carcinoma; TACE, transcatheter arterial chemoembolization; HBV-DNA, hepatitis B virus-deoxyribonucleic acid; TBIL, total bilirubin; ALB, albumin; ALT, Alanine aminotransferase; PT, Prothrombin time; NLR, neutrophil‐to‐lymphocyte ratio; PLT, platelet; AFP, alpha fetoprotein.
