# Supplementary material for: Surgical Margin Affects the Long-Term Prognosis of Patients With Hepatocellular Carcinoma Undergoing Radical Hepatectomy Followed by Adjuvant TACE
Source: Oncologist. 2023 Apr 8;28(8):e633–44. doi: 10.1093/oncolo/oyad088 (PMC10400125; doi:10.1093/oncolo/oyad088)
Supplement: oyad088_suppl_Supplementary_Table_S4 [file oyad088_suppl_supplementary_table_s4.docx]

**Supplemental online Table 4. Univariate cox regression analysis of recurrence-free survival (RFS) and overall survival (OS) in patients with wide or narrow of margin after PSM**

| **Variable** | **Wide Margin** | | | |  | **Narrow Margin** | | | |
| --- | --- | --- | --- | --- | --- | --- | --- | --- | --- |
|  | **RFS** | | **OS** | |  | **RFS** | | **OS** | |
|  | **HR (95% CI)** | ***P*** | **HR (95% CI)** | ***P*** |  | **HR (95% CI)** | ***P*** | **HR (95% CI)** | ***P*** |
| **Age**, Years  > vs. ≤60 | 0.79(0.46-1.34) | 0.375 | 0.73(0.34-1.53) | 0.400 |  | 0.96(0.59-1.56) | 0.861 | 0.85(0.48-1.51) | 0.582 |
| **Gender**,  Female vs. Male | 0.93(0.53-1.64) | 0.810 | 0.89(0.42-1.89) | 0.766 |  | 0.72(0.38-1.35) | 0.300 | 0.77(0.38-1.57) | 0.472 |
| **Diabetes**  Yes vs. No | 0.69(0.25-1.87) | 0.462 | 1.15(0.36-3.67) | 0.818 |  | 1.42(0.76-2.67) | 0.276 | 1.01(0.46-2.21) | 0.985 |
| **Child-Pugh**  B7 vs. A | 2.89(0.71-11.78) | 0.138 | 3.02(0.41-22) | 0.276 |  | - | - | - | - |
| **HBsAg**  Positive vs. Negative | 2.16(1.09-4.28) | 0.027 | 1.98(0.79-4.96) | 0.144 |  | 1.16(0.63-2.12) | 0.641 | 1.11(0.55-2.24) | 0.781 |
| **HBV-DNA level**, IU/mL  ≥ vs. <2000 | 1.03(0.64-1.66) | 0.916 | 1.48(0.81-2.71) | 0.201 |  | 1.05(0.66-1.68) | 0.829 | 0.90(0.51-1.58) | 0.716 |
| **TBIL**,μmol/L,  ≥ vs. <17 | 1.22(0.78-1.90) | 0.382 | 1.42(0.77-2.59) | 0.258 |  | 0.61(0.37-1.03) | 0.064 | 0.58(0.31-1.08) | 0.087 |
| **ALB**, g/L,  ≥ vs. < 35 | 1.95(0.48-7.91) | 0.349 | 1.71(0.24-12.38) | 0.594 |  | 0.99(0.36-2.71) | 0.992 | 1.03(0.32-3.29) | 0.956 |
| **ALT**, U/L,  ≥ vs. < 44 | 1.41(0.94-2.10) | 0.095 | 1.35(0.79-2.32) | 0.270 |  | 0.91(0.60-1.38) | 0.652 | 0.82(0.51-1.34) | 0.439 |
| **PT**, S  ≥ vs. <13 | 2.65(1.57-4.47) | <0.001 | 2.24(1.16-4.32) | 0.017 |  | 0.90(0.49-1.66) | 0.734 | 1.08(0.55-2.13) | 0.820 |
| **NLR**  > vs. ≤2.5 | 1.29(0.85-1.93) | 0.228 | 1.80(1.06-3.06) | 0.030 |  | 1.46(0.96-2.20) | 0.074 | 1.56(0.96-2.52) | 0.071 |
| **PLT**, *10^9^/ml,  > vs. ≤100 | 0.83(0.47-1.45) | 0.509 | 0.89(0.42-1.88) | 0.762 |  | 0.88(0.47-1.66) | 0.698 | 0.85(0.42-1.71) | 0.640 |
| **AFP**, μg/L,  > vs. ≤400 | 1.38(0.93-2.06) | 0.109 | 1.23(0.72-2.11) | 0.449 |  | 1.50(0.95-2.37) | 0.080 | 1.50(0.87-2.57) | 0.144 |
| **Type of operation**,  Major vs. Minor | 1.40(0.95-2.07) | 0.086 | 1.39(0.83-2.34) | 0.216 |  | 1.96(1.25-3.08) | 0.003 | 2.51(1.45-4.34) | 0.001 |
| **Transfusion**  Yes vs. No | 1.92(1.03-3.60) | 0.041 | 2.21(1.00-4.89) | 0.050 |  | 1.79(1.10-2.89) | 0.018 | 2.01(1.19-3.42) | 0.009 |
| **Tumor diameter,** cm,  > vs. ≤5 | 2.49(1.68-3.69) | <0.001 | 3.10(1.84-5.22) | <0.001 |  | 1.70(1.10-2.64) | 0.018 | 2.19(1.28-3.75) | 0.004 |
| **Microvascular invasion**  Positive vs. Negative | 2.18(1.47-3.23) | <0.001 | 3.48(2.06-5.85) | <0.001 |  | 1.87(1.24-2.82) | 0.003 | 1.97(1.22-3.19) | 0.006 |
| **Tumor capsule**,  Incomplete vs. Complete | 1.24(0.84-1.83) | 0.277 | 1.32(0.78-2.22) | 0.302 |  | 1.17(0.75-1.84) | 0.485 | 1.05(0.61-1.78) | 0.867 |
| **Edmondson-Steiner grade**,  III-VI vs. I-II | 1.40(0.91-2.14) | 0.124 | 1.90(1.02-3.52) | 0.043 |  | 1.56(0.94-2.59) | 0.083 | 2.30(1.20-4.40) | 0.012 |
| **Cirrhosis**,  Yes vs. No | 1.34(0.85-2.12) | 0.210 | 1.28(0.68-2.42) | 0.447 |  | 1.30(0.81-2.11) | 0.279 | 1.26(0.72-2.22) | 0.415 |
| **TACE**,  Yes vs. No | 0.77(0.52-1.14) | 0.194 | 0.66(0.39-1.12) | 0.124 |  | 0.64(0.42-0.97) | 0.034 | 0.59(0.36-0.96) | 0.033 |

Bold values indicate statistical significance (P < 0.05). **Abbreviation**s: OS, overall survival; PSM, propensity score matching; HCC, Hepatocellular Carcinoma; TACE, transcatheter arterial chemoembolization; HBV-DNA, hepatitis B virus-deoxyribonucleic acid; TBIL, total bilirubin; ALB, albumin; ALT, Alanine aminotransferase; PT, Prothrombin time; NLR, neutrophil‐to‐lymphocyte ratio; PLT, platelet; AFP, alpha fetoprotein.
